# Antimicrobial Susceptibility Profile and Extended-Spectrum Beta-Lactamase Phenotype of *E. coli* Isolated From Poultry

**DOI:** 10.1155/ijm/9468425

**Published:** 2025-02-22

**Authors:** Matilda Ayim-Akonor, Rita Ohene Larbi, Doreen Dela Owusu-Ntumy, Benjamin Kissi Sasu, Hilda Emefa Ohene Asa, Theophilus Odoom

**Affiliations:** ^1^Animal Health Division, Council for Scientific and Industrial Research-Animal Research Institute, Accra, Ghana; ^2^National Food Safety Laboratory, Veterinary Services Directorate, Accra, Ghana; ^3^Accra Veterinary Laboratory, Veterinary Services Directorate, Accra, Ghana

**Keywords:** antibiotics, bacteria, biosecurity, chicken, resistance

## Abstract

Bacterial resistance to antibiotics is increasing globally, with the food-animal sector (FAS) playing a key role. Knowledge of the antimicrobial resistance (AMR) of microbes from the FAS is important in the development of country-specific methods to minimize the AMR burden. In Ghana, there is limited data on the susceptibility of FAS bacteria to frequently used antimicrobials. We evaluated the susceptibility of 58 *Escherichia coli* isolates obtained from chickens to nine antibiotics and further assessed their potential to produce extended-spectrum beta-lactamase (ESBL). The Kirby–Bauer disc diffusion and combined disc methods were used following the Clinical and Laboratory Standards Institute guidelines. Nearly all isolates showed high resistance (> 50%) to all the antibiotics except gentamicin, to which more than two-thirds (*n* = 48, 83%) were susceptible. Resistance to streptomycin, tetracycline, and ampicillin was observed to be 93%, 97%, and 100%, respectively. All isolates were multidrug resistant. Over one-third of the isolates (*n* = 22, 37.9%) were resistant to seven classes of antibiotics, and a substantial proportion (*n* = 12, 20.7%) exhibited resistance to all eight antimicrobial classes. None of the isolates was detected as an ESBL producer. Most farms (86%) did not have a footbath, and the majority (71%) changed the bedding material after 4 weeks. Free-range chickens were kept on 80% of the farms. The high resistance to frequently used antibiotics suggests long-term use of these antimicrobials, which may be attributed to poor biosecurity practices that may be exposing the birds to frequent infections. There is a need to educate farmers on the prudent use of antibiotics and adherence to good biosecurity practices.

## 1. Introduction

The high demand for livestock and livestock products by humans has led to the intensification of livestock production across the globe, facilitating the spread of infectious pathogens during disease outbreaks [1]. To help prevent infections, treat diseases, and sometimes accelerate the growth of these animals, antimicrobials are often employed. It is estimated that the food-animal sector (FAS) alone consumes nearly two-thirds of the antimicrobials produced globally [2]. Since the turn of the century, there has been a global increase in antimicrobial resistance (AMR), with a devastating impact on public, veterinary, and environmental health. The World Health Organization (WHO) has classified AMR to be among the top ten global health challenges, contributing to increased treatment failure, medication costs, hospitalizations, and deaths. The greatest impact is in low- and middle-income countries (LMICs), where weak infrastructure and less efficient surveillance systems exist [3]. In the FAS, concerns about the emergence and spread of resistant bacteria extend further beyond the farm level to consumers along the value chain, increasing public health concerns [2].

The trajectory of AMR development in the FAS is particularly high in the poultry subsector where antimicrobials are mainly used for improved egg production, growth promotion, prophylaxis, and therapeutics. The misuse and abuse of antimicrobials in the global poultry sector have contributed to the continuous exposure of poultry-origin microorganisms to antimicrobials, driving the selection and spread of resistant strains. Accordingly, Van Boeckel et al. [2] reported that from 2000 to 2018, the proportion of AMR bacteria isolated from chickens increased by almost 200-fold (from 0.15 to 0.41).

*Escherichia coli* are commensals residing in the intestinal tracts of warm-blooded animals, including chickens. However, outside the intestines, some strains may become pathogenic and cause disease [4, 5]. Studies have shown that *E. coli* isolated from poultry are becoming increasingly resistant to several of the antibiotics used in the animal sector, and the production of extended-spectrum beta-lactamase (ESBL) is one mechanism being used to confer virulent characteristics to such strains, leading to treatment difficulties, higher disease outcomes, and significant economic losses [6, 7]. As a resident microbe of the intestines of chickens and a WHO indicator organism for fecal contamination of potable water, the ubiquitous nature of *E. coli* makes it an ideal candidate for the acquisition and/or dissemination of AMR genes, including ESBL, through horizontal and/or vertical gene transfer.

Chicken production is a major economic activity in the livestock sector in Ghana, providing table eggs and meat, serving as a source of employment and income generation for many along the value chain [8]. This viable economic activity employs the use of antibiotics as a disease prevention and treatment strategy during production, as happens in many LMICs, and this could drive the selection of resistant strains with ESBL phenotypes [9, 10].

To contribute to AMR knowledge and awareness in the poultry sector in Ghana, we evaluated the susceptibility of *E. coli* isolated from chickens to frequently used antimicrobials in the sector and further assessed their ESBL-producing potential as part of a surveillance study for respiratory viruses in poultry.

## 2. Materials and Methods

### 2.1. Clinical Diagnosis and Farm Selection

From April to June of 2021, birds sent by poultry farmers to the Accra Veterinary Laboratory for post-mortem examination were considered. Only birds confirmed by a veterinarian as having a respiratory infection and raised in the Greater Accra region were purposively selected for the study. The study was explained to the farmer at the clinic, and upon agreeing to participate, an informed consent was either signed or thumb-printed. The farm was subsequently visited within 24 h for sample collection. Permission for the study was obtained from the National Veterinary Epidemiology Unit and the Greater Accra Regional Veterinary Officer.

### 2.2. Sample Collection on the Farm

At the farm, the affected flock was observed, and 20 birds exhibiting respiratory signs of tracheal rales, cough, and sneezing, with or without nasal and ocular discharge, were randomly selected. The choana of each selected bird was swabbed and placed in a tube containing buffered peptone water. Samples were transported on ice to the microbiology laboratory of the Council for Scientific and Industrial Research-Animal Research Institute.

#### 2.2.1. Isolation and Identification of *E. coli*

All the samples were incubated at 37°C overnight, plated onto Sorbitol MacConkey agar (Difco, Sparks, USA/France, Claix). Using morphological characteristics, two colonies suggestive of *E. coli* were randomly selected and individually plated on Eosin Methylene Blue agar (Oxoid, Basingstoke, and Hampshire, UK) and incubated as previously described. Biochemical tests for positive indole and methyl red, and negative Voges–Proskauer and Simmons' citrate, were used to confirm *E. coli* isolates. Confirmed isolates were purified on Nutrient Agar at 37°C overnight, harvested, and stored at −20°C in brain heart infusion broth (Oxoid, Basingstoke, Hampshire, UK) with glycerol.

#### 2.2.2. Antimicrobial Susceptibility Testing (AST) and ESBL Screening

All isolates were subjected to AST using the Kirby–Bauer disc diffusion method. Nine antibiotics (Oxoid, Basingstoke, and Hampshire, UK)—ampicillin (10 *μ*g), amoxicillin/clavulanic acid (30 *μ*g), ciprofloxacin (5 *μ*g), gentamicin (10 *μ*g), chloramphenicol (30 *μ*g), streptomycin (10 *μ*g), tetracycline (30 *μ*g), trimethoprim–sulfamethoxazole (1.25/23.75 *μ*g), and nalidixic acid (10 *μ*g)—were used. Results were interpreted according to the Clinical and Laboratory Standards Institute (CLSI) [11] guidelines.

The combined disc diffusion method was used to screen all isolates for ESBL production. Cefotaxime (30 *μ*g) and ceftazidime (30 *μ*g) with and without clavulanic acid were used in accordance with the CLSI guidelines.

#### 2.2.3. Quality Control

Each batch of media and reagents was subjected to sterility and performance testing. *E. coli* ATCC 25922 was used as quality control for AST, and an in-house ESBL-positive *E. coli* was used for the ESBL screening test.

## 3. Data Analyses

Descriptive statistics were applied, and continuous and categorical variations were shown as frequencies and percentages using SPSS (Version 29). WHONET (Version 5.6) was used to evaluate antimicrobial susceptibility. Graphs were drawn using Microsoft Excel.

## 4. Results

**Figure 1:**
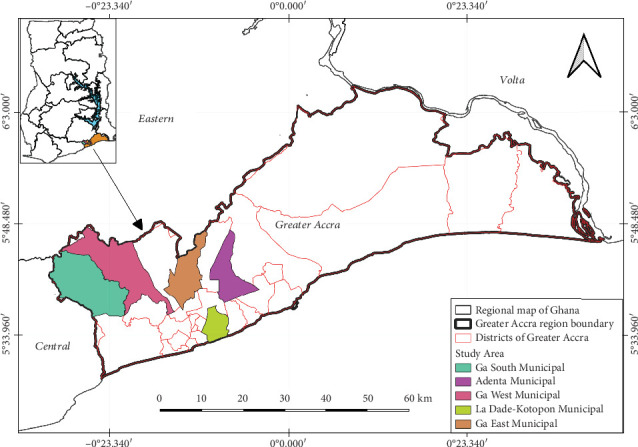
A map of the Greater Accra region highlighting the districts visited for sampling (modified from [12]).

During the study period, seven farms were successfully visited for sampling. The farms were located in 5 of the 29 districts of the Greater Accra region, namely, Ga South Municipal (two farms), Ga East Municipal (two farms), Ga West Municipal (one farm), Adenta Municipal (one farm), and La Dade-Kotopon Municipal (one farm) (Figure 1).

### 4.1. Basic Farm and Flock Characteristics

The affected flocks were predominantly layers (71.4%) and broiler birds, and their ages ranged from 5 to 35 weeks. The minimum and maximum ages of the layer birds were 20 and 35 weeks, respectively, and the majority (*n* = 3, 42.9%) of birds were 10–29 weeks old (Table 1).

Nearly all farms (*n* = 6, 85.7%) did not have a footbath at the entrance of the affected flock. The single farm with a footbath only occasionally added disinfectant. All (*n* = 7, 100%) farms practiced the deep litter system and commonly used wood shavings as bedding material. The frequency of litter change on farms varied from every 2 weeks (*n* = 2, 28.6%) to more than 4 weeks (*n* = 5, 71.4%). Most farms (*n* = 5, 71.4%) additionally kept other forms of poultry on the same premises. Of these, 60% (*n* = 3/5) kept two different types of poultry, and 40% (*n* = 2/5) kept only one type of poultry. Free-range chicken was the predominant additional poultry kept on the majority (*n* = 4/5, 80.0%) of farms, with quails and ducks occurring in equal proportion (Table 2).

### 4.2. Isolation Frequency and Antimicrobial Susceptibility

A total of 58 *E. coli* isolates were selected from the cultures of birds (two isolates per culture). Isolates were obtained from 71% (*n* = 5/7) of the farms visited. The frequency of isolation varied from a low of 15% (*n* = 3/20) in one flock, to a high of 45% (*n* = 9/20) in another flock. Two flocks each had a 30% (*n* = 6/20) frequency of isolation.

**Figure 2:**
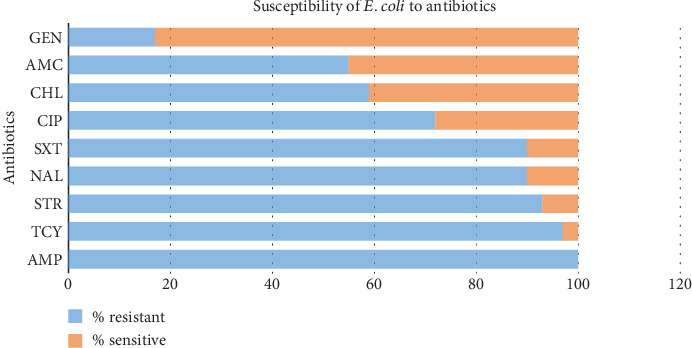
Susceptibility pattern of *E. coli* to nine antibiotics. AMP = ampicillin, TCY = tetracycline, STR = streptomycin, NAL = nalidixic acid, SXT = trimethoprim–sulfamethoxazole, CIP = ciprofloxacin, CHL = chloramphenicol, AMC = amoxicillin–clavulanate, GEN = gentamicin.

Isolates exhibited high resistance ranging from 55% to 100% against nearly all the antibiotics tested. All isolates were resistant to ampicillin. Similarly, 93% and 97% of isolates were resistant to streptomycin and tetracycline, respectively (Figure 2).

Nearly half of the isolates were susceptible to amoxicillin–clavulanate (*n* = 26, 45%) and chloramphenicol (*n* = 24, 42%), and more than two-thirds were sensitive to gentamicin (Figure 2).

The nine antibiotics used in the study belonged to eight antimicrobial classes, and the isolates showed varied susceptibility to these. All isolates exhibited multidrug resistance (MDR), defined here as resistance to three or more antibiotic classes, and were all resistant to antibiotics of at least four classes. A majority (*n* = 22, 37.9%) of isolates were resistant to seven classes of antibiotics; a substantial proportion (*n* = 12, 20.7%) of isolates were resistant to all eight classes of antibiotics (Table 3).

The antibiogram patterns of isolates showing MDR differed within and between the classes of antibiotics. The highest variability (*n* = 5) occurred among isolates that were resistant to seven classes of antibiotics, with the antibiogram pattern, TCY/AMP/CIP/STR/SXT/AMC/NAL, being the most frequently observed in more than half (*n* = 12, 54.5%) of these isolates. Isolates resistant to all eight classes of antibiotics showed two forms of antibiogram patterns, of which TCY/AMP/CIP/STR/CHL/SXT/AMC/NAL constituted the commonest (*n* = 10, 83.3%) pattern (Table 3).

## 5. Discussion

In the last two decades, surveillance of AMR in the FAS has increased tremendously. This has contributed to an increased awareness of AMR around the globe, causing some countries to reduce the quantity and/or restrict the use of certain antibiotics in the FAS as a practical means of reducing global AMR development [13]. Despite the positive outcomes in most of these countries, controlling AMR development, particularly in LMICs, will make a significant impact on the global effort, with a corresponding positive effect on public and veterinary health.

One of the useful control methods of AMR in LMICs is the adoption of practical biosecurity measures on farms as a disease-preventive measure. This will help reduce the risk of introducing pathogens on farms, the spread of disease among animals, and subsequent use of antimicrobials on farms [3]. Thus, when properly instituted, the risk of infections on farms will be reduced and the use of antibiotics in the FAS reduced.

The complete absence of functional footbaths on the farms as observed in our study suggests that animal handlers do not disinfect their footwear before entering the flock house to perform routine activities such as providing feed and water and/or collecting eggs (in the case of laying hens). It is worth noting that these activities bring the farmer to the flock house at least twice a day and could therefore facilitate the mechanical transfer of pathogens to the flock [14].

The high use of wood shavings was consistent with previous reports that identified wood shavings as the most popular type of bedding used by poultry producers across the country [9, 15]. The deep-litter system, in which poultry birds are directly placed on wood shavings which continuously get mixed with the birds' fecal droppings, spilt water, feed, and farm dust, creates a suitable environment for the survival of microorganisms including intestinal microbes [16, 17]. Therefore, the low frequency of litter change could pose an additional risk of infection to the flock.

The presence of other poultry, particularly free-range chickens and ducks that roam freely on the compound of the majority of farms, further presents an infectious disease risk to the flock. Such poultry are known to be reservoirs and carriers of immune-suppressive pathogens such as Newcastle disease virus and low-pathogenic avian influenza virus. This poses a constant threat to the flock [16, 18]. Thus, the biosecurity lapses on farms could influence farmers to use antimicrobials more often as prophylaxis which could induce selective pressure for resistant strains.

The high resistance of the isolates particularly to ampicillin, tetracycline, and streptomycin was expected as these are readily available on the market and frequently used by farmers in the country. According to Boamah, Agyare, and Odoi [9], the poultry sector is constantly threatened by diseases such as colibacillosis, enteritis, and chronic respiratory diseases. To prevent and treat these, farmers use antibiotics belonging to the tetracycline, penicillin, aminoglycoside, and fluoroquinolone classes. However, unlike developed countries such as Germany, where antibiotics for farmed animals are prescribed by a veterinarian and further administered under strict supervision [19], the situation is not the same in Ghana. With limited veterinarians in the country, farmers have, for several years, relied largely on their own experience and that of their fellow farmers in choosing and purchasing drugs and additionally performing the administration duties themselves or delegating farm attendants to do so. Thus, the frequent use of these classes of antibiotics on farmed animals by nonveterinarians could lead to overdosing or underdosing, and accelerate AMR development [10, 19]. Subsequently, resistance of commensal *E. coli* isolated from poultry fecal samples to some clinically important antimicrobials increases. For instance, resistance to tetracycline has been detected to be from 82% to 96% [20–22], identical to the 97% detected in our study, and compares favorably with the 95% detected in Egypt [23]. Similarly, complete resistance to streptomycin was reported in [22]. This compares favorably with the 93% detected in the present study. Resistance to ampicillin, of the penicillin class, has been reported to be 79% and 85% according to Mensah et al. [21] and Ohene Larbi et al. [22]. However, we observed a relatively higher resistance of 100%, which agrees with that reported by Donkor, Newman, and Yeboah-Manu [24].

Of interest is the high susceptibility of isolates to gentamicin, an observation similar to that reported in [20]. This high susceptibility to gentamicin could be because gentamicin is not a common component of the antibiotics often used by farmers in the country for prophylaxis or treatment of infection and therefore exposure of bacteria to gentamicin is very low [9].

The high MDR exhibited by all the *E. coli* isolates in the present study, with nearly one-half showing resistance to seven classes of antibiotics, and a similar rate reported in poultry over a decade ago by Donkor, Newman, and Yeboah-Manu [24] may be attributed to the long-term use of these antibiotics in the poultry sector in the country. These antibiotic-resistant commensals could present a challenge to veterinarians if involved in disease infection. Additionally, such microbes could disseminate their resistance genes to other microorganisms, accelerating AMR development in the animal sector with subsequent dissemination to humans along the food value chain.

None of the *E. coli* identified in the present study produced ESBL enzymes despite their high resistance to several antibiotic classes. Our zero ESBL finding is contrary to the relatively high prevalence of 29% reported in broilers from a rural community in the Ashanti region of Ghana [25]. The difference could be attributed to the laboratory method we employed to screen for ESBL producers. We tested two isolates per sample, whereas these authors added an ESBL-selective supplement to the culturing media, enhancing the probability of detecting ESBL producers in the entire sample.

*E. coli* isolated from the choana of poultry showed high resistance to most of the antibiotics commonly used by poultry farmers in the country. The frequent use of these antibiotics in the livestock industry could be the driving force behind this high MDR. Poultry farmers must be encouraged to observe good biosecurity practices on farms to minimize their use of antimicrobials as compensation for poor management practices.

## Figures and Tables

**Table 1:** Basic characteristics of the affected flocks in the Greater Accra region (April–June 2021).

**Parameter**	**n** ** (%)**
Flock type	
Layer	5 (71.4)
Broiler	2 (28.6)
Age	
< 10	2 (28.6)
10–29	3 (42.9)
30–39	2 (28.6)

**Table 2:** Farm husbandry practices of the affected farms in the Greater Accra region (April–June 2021).

**Variable**	**n** ** (%)**
Presence of footbath	
Yes	1 (14.3)
Frequency of litter change	
Every 2 weeks	2 (28.6)
every 4 weeks	0 (0)
More than 4 weeks	5 (71.4)
Presence of other poultry on the farm	
Yes	5 (71.4)
Frequency of other poultry on farm	
Free-range chickens	4 (80.0)
Quails	2 (40.0)
Ducks	2 (40.0)

**Table 3:** MDR prevalence and antibiogram patterns of *E. coli* isolated from flocks.

**Number of antibiotic classes**	**Resistance profile**	**% isolates (** **n** = 56**)**
4	AMP/CHL/SXT/AMC	3.5
	TCY/AMP/STR/SXT	3.5

5	TCY/AMP/CHL/SXT/NAL	3.5
	TCY/AMP/STR/SXT/NAL	3.5
	TCY/AMP/STR/CHL/AMC	3.5

6	TCY/AMP/CIP/STR/SXT/NAL	3.5
	TCY/AMP/CIP/STR/CHL/NAL	3.5
	TCY/AMP/STR/CHL/SXT/NAL	6.9
	TCY/AMP/CIP/STR/GEN/SXT/NAL	10.3

7	TCY/AMP/STR/CHL/SXT/AMC/NAL	3.5
	TCY/AMP/CIP/STR/CHL/AMC/NAL	3.5
	TCY/AMP/CIP/STR/CHL/SXT/NAL	6.9
	TCY/AMP/CIP/STR/SXT/AMC/NAL	20.7
	TCY/AMP/CIP/STR/CHL/GEN/SXT/NAL	3.5

8	TCY/AMP/CIP/STR/CHL/SXT/AMC/NAL	17.2
	TCY/AMP/CIP/STR/CHL/GEN/SXT/AMC/NAL	3.5

## Data Availability

All the data from the study are presented in the manuscript.

## References

[1] Espinosa R., Tago D., Treich N. (2020). Infectious diseases and meat production. *Environmental and Resource Economics*.

[2] Van Boeckel T. P., Pires J., Silvester R. (2019). Global trends in antimicrobial resistance in animals in low- and middle-income countries. *Science*.

[3] Sulis G., Sayood S., Gandra S. (2022). Antimicrobial resistance in low- and middle-income countries: current status and future directions. *Expert Review of Anti-Infective Therapy*.

[4] Guabiraba R., Schouler C. (2015). Avian colibacillosis: still many black holes. *FEMS Microbiology Letters*.

[5] Ibrahim R. A., Cryer T. L., Lafi S. Q., Basha E.-A., Good L., Tarazi Y. H. (2019). Identification of *Escherichia coli* from broiler chickens in Jordan, their antimicrobial resistance, gene characterization and the associated risk factors. *BMC Veterinary Research*.

[6] Badr H., Reem M., Reda N. M. (2022). Multidrug-resistant and genetic characterization of extended-spectrum beta-lactamase-producing *E. coli* recovered from chickens and humans in Egypt. *Animals*.

[7] Martínez-álvarez S., Sanz S., Olarte C. (2022). Antimicrobial resistance in *Escherichia coli* from the broiler farm environment, with detection of SHV-12-producing isolates. *Antibiotics*.

[8] Ghana Statistical Service (2020). *2017/18 Ghana Census of Agriculture: National Report*.

[9] Boamah V. E., Agyare C., Odoi H. D. A. (2016). Antibiotic practices and factors influencing the use of antibiotics in selected poultry farms in Ghana. *Journal of Antimicrobial Agents*.

[10] Nkansa M., Agbekpornu H., Kikimoto B. B., Chandler C. I. R. (2020). *Antibiotic Use Among Poultry Farmers in the Dormaa Municipality, Ghana. Report for Fleming Fund Fellowship Programme*.

[11] CLSI (2020). Performance standards for antimicrobial susceptibility testing. *CLSI supplement M100*.

[12] Donkor E., Kelly M., Eliason C. (2021). A Bayesian spatio-temporal analysis of malaria in the Greater Accra region of Ghana from 2015 to 2019. *International Journal of Environmental Research and Public Health*.

[13] Hassan I. Z., Qekwana D. N., Naidoo V. (2024). Prevalence of colistin resistance and antibacterial resistance in commensal *Escherichia coli* from chickens: an assessment of the impact of regulatory intervention in South Africa. *Veterinary Medicine & Science*.

[14] Davies R., Wales A. (2019). Antimicrobial resistance on farms: a review including biosecurity and the potential role of disinfectants in resistance selection. *Comprehensive Reviews in Food Science and Food Safety*.

[15] Ayim-Akonor M., Krumkamp R., May J., Mertens E. (2020). Understanding attitude, practices and knowledge of zoonotic infectious disease risks among poultry farmers in Ghana. *Veterinary Medicine and Science*.

[16] Chen Z., Jiang X. (2014). Microbiological safety of chicken litter or chicken litter-based organic fertilizers: a review. *Agriculture*.

[17] Plumblee Lawrence J. R., Cudnik D., Oladeinde A. (2022). Bacterial detection and recovery from poultry litter. *Frontiers in Microbiology*.

[18] Hassan M. M., Islam A., Hasan R. B. (2020). Prevalence and distribution of avian influenza viruses in domestic ducks at the waterfowl-chicken interface in Wetlands. *Pathogens*.

[19] Gross S., Roosen J., Hennessy D. A. (2023). Determinants of farms’ antibiotic consumption–a longitudinal study of pig fattening farms in Germany. *Preventive Veterinary Medicine*.

[20] Akenten C. W., Ofori L. A., Khan N. A. (2023). Prevalence, characterization, and antimicrobial resistance of extended-spectrum Beta-Lactamase-ProducingEscherichia colifrom domestic free-range poultry in Agogo, Ghana. *Foodborne Pathogens and Diseases*.

[21] Mensah G. I., Adjei V. Y., Vicar E. K. (2022). Safety of retailed poultry: analysis of antibiotic resistance in *Escherichia coli* from raw chicken and poultry fecal matter from selected farms and retail outlets in Accra, Ghana. *Microbiology Insights*.

[22] Ohene Larbi R., Ofori L. A., Sylverken A. A., Ayim-Akonor M., Obiri-Danso K. (2021). Antimicrobial resistance of *Escherichia coli* from broilers, pigs, and cattle in the Greater Kumasi Metropolis, Ghana. *International Journal of Microbiology*.

[23] Abdel-Rahman M. A. A., Hamed E. A., Abdelaty M. F. (2023). Distribution pattern of antibiotic resistance genes in *Escherichia coli* isolated from colibacillosis cases in broiler farms of Egypt. *Veterinary World*.

[24] Donkor E. S., Newman M. J., Yeboah-Manu D. (2012). Epidemiological aspects of non-human antibiotic usage and resistance: Implications for the control of antibiotic resistance in Ghana. *Tropical Medicine and International Health*.

[25] Falgenhauer L., Imirzalioglu C., Oppong K. (2019). Detection and characterization of ESBL-producing *Escherichia coli* from humans and poultry in Ghana. *Frontiers in Microbiology*.

